# Long-term results of trans-scaphoid perilunate fracture dislocations treated by open reduction and internal fixation

**DOI:** 10.1186/s12891-022-05748-1

**Published:** 2022-08-31

**Authors:** Xiao-Jun Yu, Shan-Xi Wang, Xin-Zhen Guo, Qi-Kun Liu, Ying-Guang Wang, Yun-Kun Qu, Hao Kang, Yuan Bao

**Affiliations:** 1 Department of Orthopedics, Tongji Hospital, Tongji Medical College, Huazhong University of Science and Technology, Qiaokou District, No. 1095, Jiefang Avenue, Wuhan, 430030 Hubei Province China; 2 Yantai Affiliated Hospital of Binzhou Medical College, Yantai, 264000 People’s Republic of China

**Keywords:** Dislocation, Open reduction, Perilunate, Trans-scaphoid, TSPFD

## Abstract

**Purpose:**

The paper holds the research purpose of confirming the long-term results of trans-scaphoid perilunate fracture dislocations (TSPFD) under the treatment of open reduction and internal fixation.

**Methods:**

Anteroposterial-lateral radiographs of the patient's wrist were taken before and after surgery. We use a dorsal approach for all cases. Postoperative clinical and radiographic assessments were performed routinely. The scapholunate angle (SLA), estradiol angle (RLA), as well as lunotriquetral distance (LTD) assisted in the radiographic assessment. Clinical assessment was performed using the Krimmer score, modified Mayo wrist score (MWS), active flexion extension arc (FEA), radial deviation and ulnar deviation arc (RUDA) and grip strength. A visual analog scale (VAS) assisted in the pain evaluation, the VAS score ranges from 0 to 10.

**Results:**

Twenty-two TSPFD patients due to the wrist trauma received operative treatment and we retrospectively analyzed the surgical results, together with evaluating their clinical and radiological follow-up. These patients held a mean age of 30 years old. Herzberg’s perilunate fracture-dislocation classification was taken into account to find that 19 males and 3 females suffered dorsal dislocation. The fellow-up time lasted 98.3 months on average. All cases obtained sufficient union after open reduction and internal fixation. The last follow-up found the median of grip strength was 20.00 (interquartile range, 20.00–21.25), which was 84.5% of the normal side. The modified Mayo wrist score evaluation scale considered 12 cases as excellent, and 10 good. The median of VAS and Krimmer scores at the final follow-up were 1.50 (interquartile range, 0.75–2.00) and 100.00 (interquartile range, 100.00–100.00), respectively, higher relative to the pre-operation (*P* < 0.001). No patients showed nerve damage preoperatively or postoperatively, or pin tract infection in any of the patient.

**Conclusions:**

It is necessary to diagnose such complicated biomechanical damage in early stage and adopt the open reduction and stable fixation for treatment; appropriate treatment can contribute to a functionally adequate and anatomically integrated wrist.

## Introduction

Trans-scaphoid perilunate fracture-dislocation (TSPFD) occurs infrequently, mainly due to the high-energy trauma. TSPFD, as a fracture-dislocation, seriously damages carpal anatomical structure. Perilunate dislocations take account 7% of carpal area injuries [1]. Dorsal TSPFD takes account 65% of acute perilunate dislocations [2].

The perilunate dislocations due to serious isolated ligament injuries sees the dislocation of the capitate and remainder of the carpus around the lunate along the direction of dorsal or palmar. The dorsal perilunate dislocation takes account 90% of all types of dislocation. Connection rupture between the lunate and the radius can be found in rare cases, together with the dorsal or palmar dislocation of the lunate bone, but the connection between the radius and the other carpal bones was intact, which we call the lunate dislocation. Among scaphoid fracture patients, 60% suffer perilunate or lunate dislocations, i.e.perilunate fracture-dislocation. TSPFD patients usually have radial styloid, capitatum, or triquetrum fractures [2]. If the patient has other complicated injuries or presents with inadequate radiological images, TSPFD is easily misdiagnosed, thus affecting the prognosis of the operation. The injury’s mechanism is mainly due to violence in the dorsal extension of the wrist, leading to dorsal dislocation of the carpus around the lunate and scaphoid fracture, while the anatomical position relationship between the lunate and the radioulnar joint remains unchanged [3]. However, palmar dislocations are rarely encountered. These injuries should be treated early for preventing complications, like chronic carpal instability and eventual posttraumatic arthritis. Therefore, immediate open reduction and internal fixation are necessary following closed reduction failure. The treatment could consist of open reduction, ligament repair, limited wrist arthrodesis, internal fixation, and a proximal row carpectomy may present as the final remedial measure considering the operation time and any other pathological results [4].

## Patients and methods

### General data

The study has obtained the approval of the Ethics Committee of our institution. The records of all TSPFD patients receiving open reduction and internal fixation between July 2008 and September 2016 underwent retrospective review. The study excluded those who fulfilled the criteria of: (1) misdiagnosed TSPFD initially; (2) major central or peripheral nervous system injury when injury happened; and (3) the involved wrist underwent surgery previously.

The current study analyzed the union rates as well as the clinical and radiological results regarding the open reduction and fixation in patients with TSPFD during long-term follow-up. The authors hypothesized that open treatment would achieve a good effect.

Preoperative and postoperative wrist anteroposterior (AP) and lateral (LAT) radiographs were employed for evaluating all these cases. The modified Herzberg’s perilunate fracture-dislocation classification [5] classified all the cases dorsal type. All the cases exhibited scaphoid body fractures.

### Surgical technique

**Fig. 1:**
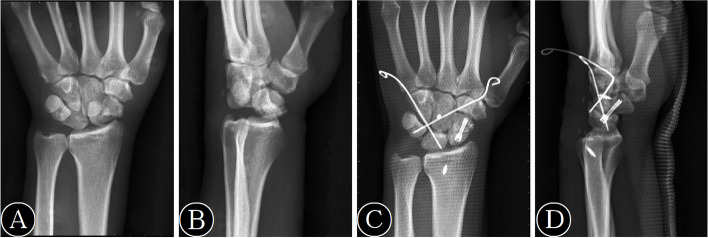
A 54-year-old man with right TSPFD. **A** Preoperative anteroposterior X-ray. **B** Preoperative lateral X-ray. **C** Postoperative anteroposterior X-ray. **D** Postoperative lateral X-ray

All cases were all done by the same hand surgical team at the same hospital, and the average age of surgery is 10 years. We used brachial plexus anesthesia. Second—generation non—steroidal analgesics are used for postoperative analgesia. The average time of intraoperative tourniquet use was about 1 h. We used a dorsal approach and made a 3 cm longitudinal incision dorsally following Lister’s tubercle. The surgeon raised the flaps ulnarly and radially, and the incision went down to the extensor retinaculum. The surgeon divided the retinaculum according to the third dorsal compartment, and identified the extensor pollicis longus tendon distally with radial retraction. It was only allowed to see the release of the distal 1 cm of the third compartment. Then, it was possible to see reflection of the second and fourth compartments off the dorsal capsule. Then the surgeon developed a capsulotomy and extended it longitudinally. After removing the bone or cartilage fragments from the intercarpal joint, the surgeon irrigated the joint for removing any hematoma or other debris. The surgeon reduced scaphoid fracture with K-wires, and introduced the Herbert screw fixation guidewire along the long axis of the scaphoid, followed by inserting a Herbert mini screw over the guidewire after reaming. Then, the surgeon reduced the lunotriquetral joint and pinned percutaneously from the ulnar side, followed by using K-wires to stabilize the perilunate joint. The surgeon used absorbable 2–0 or 3–0 sutures to close the capsulotomy incision interruptedly. A seek nail was used to fix the retinaculum, and nylon sutures were used to close the skin. When the operation was completed, the surgeon placed a sugar-tong splint, to make the wrist and forearm in the neutral position [6] (Fig. 1).

### Postoperative management

We suggested all patients to undergo digital exercises immediately for reducing swelling. After discharge, all patients were followed up with at 2 weeks, 1 month, 2 months, 3 months, 6 months and 12 months post-operatively and then annually till the final follow-up. At the first postoperative follow up 2 weeks after surgery, patients were required to take a well-molded short arm cast for holding the wrist in a functional position for 6 to 12 additional weeks, the functional position refers to the dorsal extension of the wrist about 20–25 degrees, that is, the position of the wrist joint when the palm is firmly grasped, the thumb is fully extended, and the metacarpophalangeal and interphalangeal joints are slightly flexed. 6–8 weeks after the operation, patients did not need to wear the short arm cast or K-wires, and they were encouraged to perform active-assisted wrist movement exercises.

Fracture nonunion is a common complication of fracture prognosis, fracture union is determined as absence of fracture line and continuous callus formation on X-ray. To make sure there are no such complications, postoperative clinical and radiographic assessments were performed by two surgeons who were not participated in the treatment of patients at every follow up.

After the fixation material is removed, the patient is instructed to perform physical rehabilitation to prevent muscle atrophy and exercise the range of motion of the hand and wrist. They were allowed to take sports and various activities after 5 to 6 months [7]. And our patients should carry out rehabilitation training every day, generally 5 to 6 months after the operation can return to normal work.

Postoperative clinical and radiographic assessments were performed after discharge, all patients would be followed at 2 weeks, 1 month, 2 months, 3 months, 6 months and 12 months postoperatively, and then at annually until the last follow-up. The scapholunate angle (SLA, normal range, 30–60°), radiolunate angle (RLA, normal average, 2°), and lunotriquetral distance (LTD, normal range, 1.3–2.2 mm) were used for radiographic assessment. Postoperative clinical and radiographic assessments were performed by two surgeons who were not participated in the treatment of patients at every follow up. Clinical assessment was performed using the Krimmer score [8], modified Mayo wrist score (MWS) [9], active flexion extension arc (FEA), radial deviation and ulnar deviation arc (RUDA) and grip strength [10], we use a triangle ruler to measure the range of motion of the joint, and we use a grip meter to measure the patients’ grip strength which is in kilograms. Krimmer score and MWS are often used in conjunction with the functional score of the hand, foot and ankle, which systematically evaluates the patient's pain, joint function, range of motion, etc. (Tables 1,2) [11]. A visual analog scale (VAS) assisted in pain evaluation, we used the 11-point visual analogue scale. The average of these scores at the last follow-up was calculated for comparison.

**Table 1:** Krimmer Score

Grip trength(% of contrlateral)	Percentage(%)			
	0–25			0
	> 25–50			10
	> 50–75			20
	> 75–100			30
Range of motion	extension/flextion	ulnar/radial abduction	pronation/supination	
	< 30	< 10	< 80	0
	31°-69°	10°-35°	81°-110°	10
	61°-100°	36°-50°	111°-140°	15
	> 100	> 50°	> 140°	20
Pain	Degree			
	Severe			0
	With/without stress			10
	Only with stress			15
	pain free			20
Restrictions	Degree			
	Severe			0
	Fair			10
	Only with certain activities			20
	Normal, no restrictions			30

**Table 2:** Mayo wrist score

Pain	VAS score	Degree of pain	Points
	0	No pain	25
	1–4	Mild, occasional	20
	5–7	Moderate, tolerable	15
	8–10	Severe to intolerable	0
Functional status		Details	Points
		Return to regular empoyment	25
		Restricted employment	20
		Able to work, unemployed	15
		Unable to work, pain	0
Range of motion	Total motion	Percentage of normal(%)	Points
	≧20°	90–100	25
	100°-119°	80–89	20
	90°-99°	70–79	15
	60°-89°	50–69	10
	30°-59°	25–49	5
	0° = -29°	0–24	0
Grip strength		Percentage of normal(%)	Points
		90–100	25
		75–89	15
		50–74	10
		25–49	5
		0–24	0
91–100––-Excellent	80–90––-Good	65–79––-fair	< 65––-Poor

### Statistical analysis

Statistical analyses were performed using Statistical Package for the Social Sciences (SPSS 20.0, IBM, New York City, USA). Categorical data were tabulated as frequencies or percentages. Normally continuous data were expressed as the mean values and ranges, and non-normally continuous data were expressed as the median and interquartile range. Normality was tested using the Kolmogorov–Smirnov test. Fisher’s exact probability test (n < 40) for categorical variables was used to compare patients’ characteristics at baseline. The paired t test was used to analyze intergroup comparisons for normally distributed continuous data. A P value of less than 0.05 was considered statistically significant.

## Results

The study included 22 acute (< 1 week) TSPFD patients in total who had open reduction and internal fixation. All clinical data was analysed retrospectively. The mean follow-up time was 98.4 months (range, 49–159 months). Patients held a mean age of 32 years old (range, 15–58 years). We evaluated their clinical and radiological follow-up. Nineteen patients suffering a dorsal dislocation were males, and 3 were females. A detailed analysis indicated that 5 cases were injured during sports activities, 3 cases fell from a high place and 14 cases were injured due to simple falls (Table 3).

**Table 3:** Base data of the patients with TSPFD

Patient No	Gender	Age (years)	Side	Causes
1	Male	20	Left	Falling from height
2	Male	54	Right	Tumble
3	Male	49	Right	Tumble
4	Male	30	Left	Tumble
5	Female	55	Left	Tumble
6	Male	18	Left	Tumble
7	Male	58	Right	Falling from height
8	Male	35	Right	Tumble
9	Male	21	Left	Sport injury
10	Male	30	Right	Tumble
11	Female	21	Left	Sport injury
12	Male	18	Right	Sport injury
13	Male	29	Left	Tumble
14	Male	31	Left	Tumble
15	Male	37	Left	Tumble
16	Male	25	Right	Tumble
17	Male	15	Left	Sport injury
18	Male	30	Left	Tumble
19	Male	20	Right	Tumble
20	Female	43	Right	Falling from height
21	Male	22	Left	Sport injury
22	Male	34	Left	Tumble

For all patients, the incision healed primarily, and didn’t show infection. The hospital stay average length was 10.0 days (range, 5–18 days). Postoperative radiographic evidence showed that all these cases got sufficient union, and average healing time was 13.2 weeks (range, 10–15 weeks). The median of postoperative SLA, RLA, and LTD were 50.00° (interquartile range 49.00–51.00°), 1.90° (interquartile range, 1.80–2.00°) and 2.10 mm (interquartile range, 1.90–2.20 mm), respectively. Reduction loss, pseudarthrosis, or avascular necrosis did not happen (Table 4).

**Table 4:** Clinical outcomes of 22 cases

Patient	Hospital day	Union time	Follow-up	SLA	RLA	LTD	Krimmer score		VAS score		At last follow-up				Complications
No	(days)	(weeks)	(months)	(°)	(°)	(mm)	Preop	Final	Preop	Final	MWS	FEA	RUDA	Grip strength	
1	11	10	159	48	2	2.1	50	100	6	2	E	122	36	25	none
2	7	12	145	49	2	2.2	45	100	6	2	G	117	36	20	none
3	10	14	133	50	2	2.2	35	95	7	3	G	119	38	20	none
4	13	15	134	48	1.8	2.1	40	100	5	2	E	112	37	20	none
5	10	13	140	49	1.9	2.3	40	100	5	2	E	115	35	25	none
6	14	14	124	49	1.9	2.1	40	100	5	2	G	119	35	20	none
7	11	15	124	50	1.9	2.3	45	100	5	2	E	121	34	25	none
8	6	14	128	51	1.8	2	40	100	6	2	E	120	35	20	none
9	18	15	94	51	1.8	2	35	100	7	3	G	121	37	20	none
10	10	14	95	51	2	2.1	45	100	6	1	E	119	37	20	none
11	6	13	95	49	2	1.9	45	100	6	1	E	120	37	20	none
12	7	12	85	49	2	1.9	45	95	6	3	G	123	35	20	none
13	11	12	86	49	2	1.9	55	100	5	0	E	123	35	20	none
14	10	13	87	49	2	1.8	55	100	5	0	E	123	34	25	none
15	8	14	77	50	2	2.2	35	100	7	1	G	120	35	20	none
16	9	14	77	50	1.9	1.9	50	100	6	1	G	118	35	20	none
17	9	15	77	50	2.1	2.1	35	100	7	0	E	124	38	20	none
18	13	11	54	50	1.8	2.2	40	100	6	0	G	120	38	20	none
19	5	14	52	51	1.7	1.9	55	100	5	1	E	121	36	25	none
20	15	13	49	51	1.7	1.9	45	100	5	2	G	121	35	20	none
21	6	12	91	51	1.7	2.1	55	100	5	0	E	122	35	20	none
22	12	12	58	49	1.8	2.2	55	100	6	1	G	122	36	20	none

Abbreviations: *SLA s*capholunate angle, *RLA* radiolunate angle, *LTD* lunotriquetral distance, *VAS* visual analog scale, *MWS* modified Mayo wrist score, *FEA a*ctive flexion extention arc*, RUDA* radial deviation and ulnar deviation arc

At the final follow-up, the modified Mayo wrist score defined 12 cases as excellent, and 10 cases good. The mean FEA was 120.09° (range, 112–124°), and the median of RUDA was 35.50° (interquartile range, 35–37°). The median of grip strength was 20.00 (interquartile range, 20.00–21.25), which was 84.5% of the normal side. After the operation, patients presented better flexion, extension, FEA and RUDA at the last follow-up. Midcarpal arthritis on radiographs did not happen at the final follow-up. (Table 4).

The median of VAS and Krimmer scores at the final follow-up were 1.50 (interquartile range, 0.75–2.00) and 100.00 (interquartile range, 100.00–100.00), respectively. The VAS and Krimmer scores were significantly better than those preoperatively, which were 6.00 (interquartile range, 5.00–6.00) and 45.00 (interquartile range, 40.00–51.25), respectively (*P* < 0.001) (Table 5). A typical case is shown in Fig. 1.

**Table 5:** Comparison of clinical outcomes between preoperation and final follow-up

	Pre-opertation	Final follow-up	*P* value
Krimmer score	45.00 (40.00—51.25)	100.00 (100.00—100.00)	< 0.001
VAS score	6.00 (5.00—6.00)	1.50 (0.75—2.00)	< 0.001

Abbreviations: *VAS* visual analog scale

## Discussion

Because patients with TSPFD are extremely rare, medium- and long-term follow-up studies on such patients are few. In addition, the data on the effect and prognosis of open surgery also need to be analyzed and compared among a large number of patients. A long-term follow-up was conducted in the study regarding 22 TSPFD patients.

The arthrodesis was put forward by Wagner in 1959 for TSPFD treatment, when closed reduction could not work well; in 1965, Campbell put forward the proximal row carpectomy; and in 1944, MacAusland recommended the open reduction and lunate excision for patients diagnosed within and later than 6 weeks, respectively [12]. The open reduction and internal fixation were also mentioned even in delayed cases [13]. For cases with acute TSPFD caused by scaphoid fracture, reduction loss and instability are of common occurrence, even after the achievement of closed reduction [14]. In the study by Adkison and Chapman, for TSPFD patients under closed reduction and cast immobilization, there was a 59% reduction loss in 6 weeks [15]. Such kind of patients should take traction and reduction maneuvers in emergency rooms to relieve and reduce pain. Nevertheless, they are difficult to complete, particularly under the situation of lunate rotation and palmar dislocation. These maneuvers could also aggravate the palmar radiolunate ligament injuries [15]. Open reduction can assist in evaluating the injury, reduction and repair [13]. Therefore, the surgical approach was chosen.

Common surgical approaches include dorsal, volar and combined approaches. The experience of surgeon and injury type shall be considered for decision making [4]. We did not use the palmar approach. We believe that the volar approach affects the flexion of the wrist, especially when scar tissue is present. In addition, the dorsal approach makes it easier to expose the surgical area in a minimally invasive environment.

Herbert screws was first invented as scaphoid fracture in the 1990s, and now can serve for successfully resolving irritation and motionlessness problems of K wires [16, 17]. Herbert screws were used in all cases for the fixation of scaphoid fractures. We observed no reduction loss or nonunion.

In literature, we can find different reports regarding the surgical treatment of such injuries. Oh WT The surgeon performed arthroscopic K-wire reduction in 5 patients with TSPFD, reporting 7 excellent, 8 good, and 5 fair outcomes based on the MWS [6]. Open reduction, ligament repair, and K-wire fixation were performed in six cases, reporting 1 excellent, 2 moderate, and 3 poor outcomes according to the Green and O’Brien evaluation scale [13]. The study obtained 12 excellent, 10 good, and 0 fair outcomes according to the MWS, so we believe that our method of operation is advisable.

Komurcu et al. found that effective emergency management resulted in more satisfactory outcomes, although the correlation between radiographic results and clinical outcomes was not always consistent [18]. 8 perilunate fracture-dislocation cases were examined in the study by Cemal K, finding the influence of injury type, injury severity, perilunate dislocation type, and time to diagnosis on clinical and radiological results [12]. Additional pathologies decided the necessity of using postoperative cast immobilization. The cast immobilization duration is different for different people, but It is determined by scaphoid fixation and interarticular stability of the carpal bone. The treatment time is in the range of 6–12 weeks in the literature, and shall be limited in 6 weeks if there is a good stabilization [19]. The cast was in place for 6, 8 and 12 weeks for 9, 12, and 1 of our patients, respectively.

There are several limitations in our study. First, no control group that uses different treatment methods is adopted in the study, hence a general conclusion can only be made by comparing with previous similar studies. Second, the sample size is small. Finally, it is a retrospective and observational study. However, our study’s strength is its relatively long follow-up period compared to similar studies.

## Conclusion

To sum up, such type of injury is connected with complicated biomechanical damage to wrist anatomy. If we can diagnose TSPFD, more found in the young population, in early stage as well as treat it by the open reduction and stable fixation, it is possible to obtain functionally sufficient wrist with anatomical integration.

## Data Availability

People are not allowed to publicly obtain the datasets that are developed and/or analyzed in the study affected by ethical approval limitations that involve patients’ data and anonymity, but can obtain from the corresponding author with permission.
